# Stressing State Analysis of Partially Prestressed Concrete Beams with High Strength Reinforcement Based on NSF Method

**DOI:** 10.3390/ma15093377

**Published:** 2022-05-08

**Authors:** Jian Yuan, Feng Xu, Heng Du, Suhui Yu, Guorui Sun

**Affiliations:** 1 Wuhan Institute of Technology, School of Civil Engineering and Architecture, Wuhan 430205, China; yuanjian_850809@126.com (J.Y.); du970609@163.com (H.D.); 2 Academy of Combat Support, Rocket Force University of Engineering, Xi’an 710025, China; yusuhui88@126.com; 3 School of Transportation Science and Engineering, Harbin Institute of Technology, Harbin 150090, China; 19b933012@stu.hit.edu.cn

**Keywords:** concrete, reinforcement, beam, stress, analyses, mutation, interpolation

## Abstract

This paper analyzes the flexural behavior of a partially prestressed steel high-strength reinforced concrete beams based on the structural stress state theory and the numerical shape function method. First, the generalized strain energy density is formed by the measured strain data of the test beam to reflect the structural stress state of the beams, and then the Mann–Kendall criterion is used to judge characteristic points of the generalized strain energy density curve. Two characteristic points, namely, post-elastic boundary load and failure load, are detected, so that the whole loading process is divided into three structural stressing state stages. Unlike the ultimate load, failure load is defined according to the general law from quantitative to qualitative change, which represents the starting point of the failure stage of the beam. Then, experimental strains and deflections, strain/stress fields interpolated by the numerical shape function method, and internal forces calculated by integration are respectively analyzed to obtain their changing characteristics and working behavior around the characteristic points, which can also verify the correction and effectiveness of the Mann–Kendall criterion. In addition, through the analysis above, it can be known that the failure loads of the test beams can be effectively improved by increasing the prestressed reinforcement ratio or concrete strength.

## 1. Introduction

Compared with ordinary reinforcement, high-strength reinforcement has the following advantages: high performance, good ductility, and high purity [1], which can not only improve the bearing capacity of members but also reduce the number of the reinforcement in reinforced concrete members to reduce its energy consumption [2]. In addition to the application of high-strength reinforcement in residential buildings, industrial buildings and urban public buildings around cities and towns, and even some bridges also have a great demand for high-strength reinforcement, which shows the broad application prospects of high-strength reinforcement in concrete structures [3,4,5,6]. Although high-strength reinforcement is not the latest building material, with the further upgrading of reinforcement application in the construction industry, high-strength reinforcement still has research value.

Compared with ordinary reinforcement, high-strength reinforcement has better mechanical properties. Among them, Qin et al. [7] conducted uniaxial tensile tests on HS600 steel bar under different strain rates, and the results showed that HS600 steel bar has significant strain rate sensitivity. Elices et al. [8] conducted a fracture test on SBS steel bars to analyze brittle fractures of high-strength steel bars under considering the plane strain fracture toughness. Compared with ordinary reinforcement, Han’s [9] tensile test of UHS reinforcement shows that UHS reinforcement has higher strength, shorter yield platform, lower ductility, and a more obvious post ultimate stress zone. Shahrooz et al. [10] reassessed to evaluate the AASHTO specifications concerning the use of high-strength reinforcing steel and other grades of reinforcing steel, and then, some of his recommendations were incorporated into the 2013 interim revisions of the specifications.

With the gradual increase in research on high-strength steel bars, researchers began to pay more attention to the high-strength reinforcement concrete members. To study the influence of high-strength reinforcement on the flexural behavior of concrete beams, researchers carried out a series of related experimental studies around the configuration of high-strength reinforced concrete beams [11,12]. For example, Anggraini et al. [13] conducted a test on the flexural bearing capacity of reinforced concrete beams designed with high-strength reinforcement and found that high-strength reinforcement can improve the flexural performance of concrete beams by 16–18%. Juan et al. [14] conducted experiments on six deep beams with 880 MPa high-strength reinforcement to study the effect of the reinforcement ratio of high-strength reinforcement on its flexural capacity. Hassan et al. [15] conducted static load tests on 12 concrete beams strengthened with high-strength steel stirrups to study their influence on the mechanical properties of concrete beams. These results show that the bearing capacity of concrete beams can be greatly improved due to the good bonding performance of high-strength reinforcement.

The high-strength reinforcement can not only improve the mechanical properties of concrete beams but also improve the deformation capacity of beams [16]. Hwang et al. [17] tested the beam-column connection strengthened with high-strength reinforcement. The results show that when high-strength reinforcement is externally connected, the reinforcement at the bottom of the beam will bond and slip, reducing the deformation capacity of the test beam. To understand the influence of different high-strength reinforcement methods on the deformation performance of concrete beams, Joon et al. [18] conducted load tests on concrete beams equipped with SD500 and SD600. The test results show that the inclined reinforced coupling beam specimen has greater ductility than the horizontal reinforced specimen. In the experimental study on the flexural performance of concrete flexural members with high-strength reinforcement, the flexural capacity, shear capacity and other mechanical properties of concrete members can not only be greatly improved but also their deformation capacity will be improved [19,20,21,22,23].

From the above literature, it can be demonstrated that the division of stress state stages of concrete flexural members with high-strength reinforcement is only determined by the load–displacement curve and load–strain curve in the experiment or simulation. In the existing experimental analysis, the measured experimental data have not been fully applied, resulting in the neglect of the hidden working characteristics of the beam in the actual analysis process, which can be extracted from the experimental data by appropriate methods. Although researchers can carry out a lot of experiments to obtain more accurate experimental conclusions, such inefficient analysis is only a waste of experimental data. However, with the help of the finite element method, the mechanical properties of the structure will be clearer. Inevitably, the traditional finite element model is usually simplified or assumed by researchers according to experience. Therefore, the corresponding results calculated by these models will deviate from the actual experimental results to varying degrees. In this case, a new finite element analysis method is needed to make full use of the collected experimental data to accurately reveal the hidden working characteristics of the beam.

Therefore, this paper presents an analysis method combining the structural stressing state theory and the numerical shape function (NSF) method to analyze the mechanical performance of high-strength reinforcement concrete members from a relatively new perspective. The theory of structural stress state is a theoretical method based on the common variation characteristics embodied in the structural working process and referring to the structural working characteristics revealed by classical mechanics. The numerical shape function is an effective interpolation method for the experimental data of any number and any position formed by the numerical simulation method. The generalized strain energy density (GSED) is used to represent the structural stressing state of the whole partially prestressed steel high-strength reinforced concrete beam, and the Mann–Kendall criterion is used to distinguish the mutation characteristics of the structural stressing state of the test beams, to redefine the failure load of the test beams. Then, the experimental strain data are interpolated by the NSF method so that the obtained strain/stress fields and internal forces can be obtained to reflect the working behavior in detail. Using the analysis method used in this paper, we will be able to analyze the stress of high-strength reinforced concrete members more intuitively and comprehensively, which will also contribute to the research and development of high-strength reinforced concrete members.

## 2. Method and Theory of Structural Stress State

### 2.1. Numerical Description of the Structural Stressing State

The theory and method of structural stress state [24,25] is a theory used to model and analyze structural response (strain, displacement, etc., of test/simulation). The characteristics of work behavior revealed by this method are the general law from quantitative change to qualitative change. The displacement of key nodes and the strain of key parts are the most direct manifestation of a structural stress state. However, displacement, strain, and other responses are directional, which has a great impact on the numerical model expression of the structural stress state.

To redefine the failure load of the structure based on the structural stress and strain, a physical parameter-generalized strain energy density (GSED) [26,27] is introduced to reflect the strain state of the whole structure.

For a measuring point of the test beam, its GSED can be expressed as

(1) $$E_{ij}=\int_{0}^{\varepsilon_{ij}}\sigma_{ij}d\varepsilon ,$$

where *E_ij_* is the GSED value of the *i*-th point under the *j*-th load. *ε_ij_* is the strain value of this point. To reflect the characteristics of the force state of the entire structure, the GSED of all measurement points is summed, that is,

(2) $$E_{j}=\sum_{i=1}^{n}E_{ij},$$

where *E_j_* is the GSED and value of each point under the action of the *j*-th load. Through the dimensionless process, the GSED and value are normalized to obtain the dimensionless physical quantity *E_j_*, norm, that is

(3) $$E_{j,\;\mathrm{norm}}=\frac{E_{j}}{E_{\max}},$$

in which *E*_max_ is the maximum GSED and value in the whole process, and *E_j,_*_norm_ of all measuring points reflects the stress state of the structure under each load step *F_j_*. In this way, a curve *E_j,_*_norm_-*F_j_* reflecting the characteristics of the stress state of the test beam is formed, and this curve can reflect the working model of the structure under the external load.

### 2.2. Application of Mann–Kendall Criterion

The Mann–Kendall trend test method is a widely used non-parametric statistical test method. It does not require the sample to follow a certain distribution and is not affected by a small number of outliers [28,29]. Therefore, this method can be applied to the *E_j_*-*F_j_* curve to determine the structure’s sudden changes in the loading process. It is supposed that the sequence of {*E_j_*(*i*)} (the *i*-th load step, *i* is 1, 2…n) is statistically independent, and a new random variable is defined at the *k*-th load step *t_k_* is

(4) $$t_{k}=\sum_{i}^{k}m_{i},\;\left(2\leq k\leq n\right),$$

(5) $$m_{i}=\left\{ \begin{array}{ll} +1\;, & E_{j}(i)>E_{j}(j),\left(1\leq i\leq j\right)\\ 0\;, & \mathrm{otherwise} \end{array}\right.,$$

Then, the average value *E*(*t_k_*) and variance *Var*(*t_k_*) of *t_k_* are calculated by the following equations:

(6) $$E(t_{k})=\frac{k(k-1)}{4},(2\leq k\leq n),$$

(7) $$Var(t_{k})=\frac{k(k-1)(2k+5)}{72},(2\leq k\leq n),$$

Assuming that {*E_j_*(*i*)} sequence is statistically independent, a new statistic *DF_k_* is defined and calculated as follows:

(8) $$DF_{k}=\left\{ \begin{array}{ll} 0, & \;\;\;\;\;k=1\\ \frac{t_{k}-E(t_{k})}{\sqrt{V_{\alpha r}(t_{k})}}, & 2\leq k\leq n \end{array}\right.,$$

When the initial sequence {*E_j_*(*i*)} is reversed, the corresponding change trend should be opposite to the original sequence. Therefore, the corresponding statistic *DF_k_* should use the opposite symbol to represent the correct trend of the inverse sequence. Thus, a new statistic *DB_k_* is calculated by Equation (8) above. Hence, *DF_k_*-*F* and *DB_k_*-*F* curves are drawn in the same graph, and the intersection of the two curves is the mutation point of the structural stressing state, which is the characteristic load of *E_j,_*_norm_-*F_j_* curve.

## 3. Experimental Introduction

### 3.1. Design and Fabrication of the Test Beam

**Figure 1:**
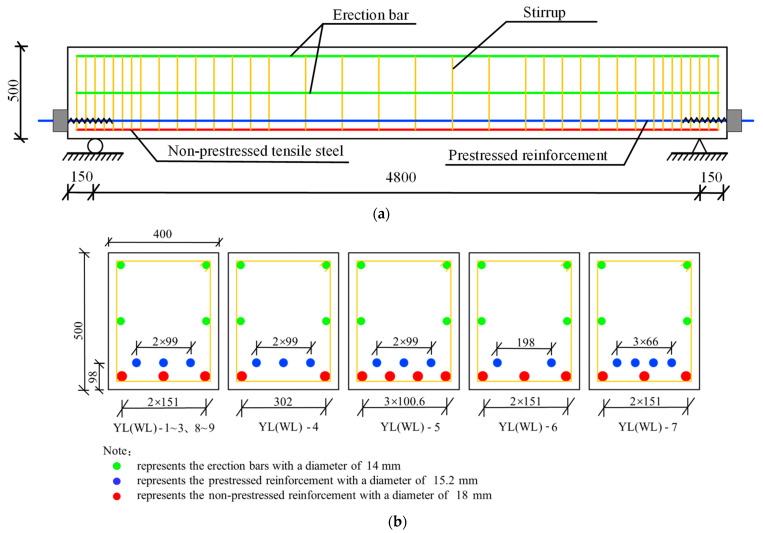
Dimensions and reinforcement layout of the test beams (Unit: mm): (**a**) dimensions of the test beams; (**b**) reinforcement layout of the test beams.

Li Qiang [30] designed and manufactured 18 simply supported partially prestressed steel high-strength reinforced concrete beams in the laboratory of Hebei University of Technology, all of which were constructed by the post-tensioned method. According to the bond formation of the prestressed steel strand, the test beams can be divided into the bonded beam and the unbonded beam. The size and the reinforcement layout of each test beam are shown in Figure 1.

The design differences of each beam are shown in Table 1.

The concrete strength grades used in this test beam are C40, C50, and C60, respectively. The tensile strengths of non-prestressed reinforcement are 400, 500 and 600 MPa, respectively. The diameter of the non-prestressed tensile reinforcement used in this test beam is 18 mm. Both the erection and stirrups are made of 400 MPa steel bars. The prestressed steel strand is 1 × 7 *φ*^s^ 15.2 (*f_ptk_* = 1860 MPa) (*φ*^s^ stands for steel strand); the relaxation grade is II; the nominal cross-section area is 140 mm^2^, and the prestressed strand wires are all single row symmetrical configuration. 

The non-prestressed tensile reinforcement used in the test was subjected to an axial tensile test, and the mechanical properties obtained from the actual test are shown in the Table 2.

It subjected the same batch of reserved sample sections of the steel strand used in the test to axial tensile tests, and the test results are shown in the Table 3.

Through the compressive strength test of the cube test block, the actual measured value of the concrete compressive strength of the test beam is obtained in the following Table 4.

### 3.2. Arrangement of Measuring Points and Loading Scheme

**Figure 2:**
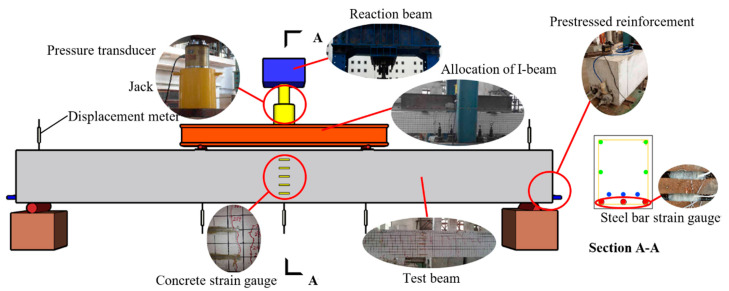
Experimental model and apparatus of the test beam.

As shown in Figure 2, this test is a static load test, and the loading method is four points bending loading. The layout of test beam measuring points is shown in the figure, and the concrete strain measuring points are named No. 1 to No. 5 from top to bottom. To check whether the test beam is in close contact with the whole loading device and whether there is an inclination, the test beam is preloaded. The preloading is carried out in two stages, and the load value of each stage is 20 kN. During the formal loading, the loading value of each level is 20 kN. When the cumulative load value is close to the calculated cracking load and yield load, the loading value of each level is reduced to 10 kN to obtain the measured characteristic load value more accurately. When the tensile non-prestressed reinforcement yields, the loading mode is changed to displacement control, and the loading displacement of each stage is 1 mm until the strain of the tensile non-prestressed reinforcement reaches about 10,000 με or the mid-span deflection of the test beam reaches 1/50 of its span [31], and the test beam fails. 

## 4. Stress State Analysis of the Test Beam

### 4.1. E_j,__norm_-F_j_ Curves and Failure Loads of Test Beams

**Figure 3:**
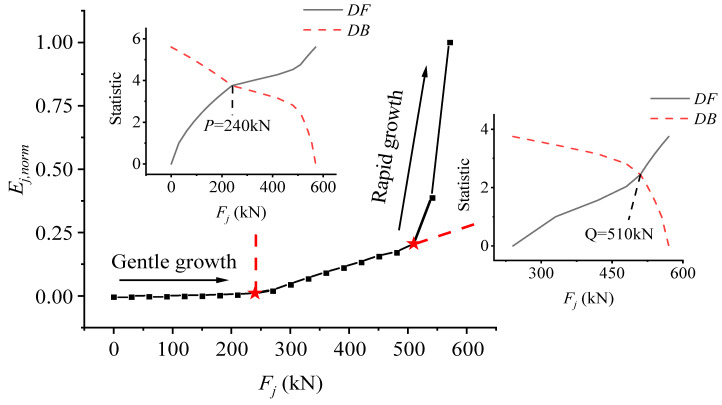
The *E_j_*_, norm_-*F_j_* and M-K statistic curves of the test beam.

Based on the proposed method and theory of the structural stress state, generalized strain energy density calculation and M-K discrimination are performed on the strain data measured in the test to determine the development trend of the structural stressing state mode of the test beams. Taking the UP4 beam as an example, the *E_j,_*_norm_-*F_j_* curve is drawn to study the development trend of stressing state of the UP4 beam, shown in Figure 3. The M-K test is used to obtain two feature points *P* and *Q* in the *E_j,_*_norm_-*F_j_* curves. As can be seen in the figure below, the *E_j,_*_norm_-*F_j_* curves present three segments before and after *P* and *Q*: before the characteristic point *P*, the curve is displayed as linear. At this stage, the test beam is in the linear elastic stage, so the *E_j,_*_norm_ at this stage does not change much. As the load continues to increase, the slope of the curve also increases. At this stage, the test beam is in the post-elastic stage, so the characteristic point *P* can be called the post-elastic boundary load. When the characteristic point *Q* is exceeded, the slope of the curve increases sharply. At this time, the working model of the test beam has changed qualitatively, which indicates that the structure begins to fail. Therefore, characteristic point *Q* can be defined as the failure load of the test beam. The failure load *Q* defined here is not the load when the structure is destroyed, but when the structure reaches the failure load, the working model of the structure changes qualitatively, and the structure gradually fails until it reaches *E*_max_, and then, it is destroyed as a whole. It should be pointed out that the failure load defined here is not the ultimate load, it manifests the starting point of the failure stage based on structural stressing state theory, which reflects the boundary point of the quantity to the quality change.

### 4.2. Reflection of Structural Stressing State Characteristics of Test Beam in Strain

Because the most unfavorable position of the structure in the simply supported beam is the mid span section, the mid span section is selected as the control section of the simply supported beam to explore the variation characteristics of the stress state of the structure. To reflect the strain development of each measuring point in the mid-span section of the test beam, the strain diagram of the UP4 beam is drawn, and the ultimate strains of concrete (345 με) and yield strain of steel bar (3271 με) are also marked in the diagram.

**Figure 4:**
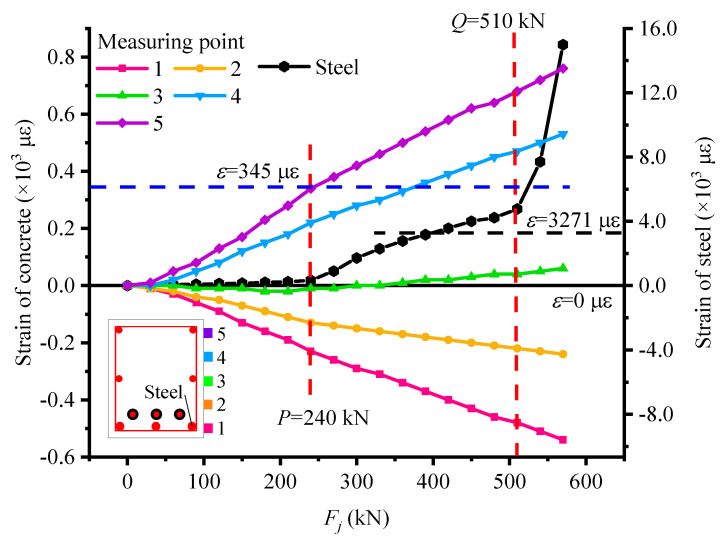
Strain diagram of unbonded No. 4 beam (UP4).

As shown in Figure 4, when the applied external load is close to the characteristic load *P*, measuring point 5 of the UP4 beam at the bottom edge of the mid-span section reaches the ultimate tensile strain, and measuring point 4 reaches the ultimate tensile strain between loads *P* and *Q*. This is because, with the increase of applied load, the tensile strain at the bottom edge gradually increases until the ultimate tensile strain of concrete is reached. Then, the bottom edge begins to crack, and the cracks gradually develop upwards. In addition, before load *P,* the strain curve of non-prestressed steel bars is approximate to a straight line, and the strain increase is tiny, which indicates that the beam is in the stage of linear elasticity, and the beam enters the post-elastic stage from the linear elastic stage. When overloading P, the number and width of cracks increase, and the bottom concrete gradually stops working, resulting in the non-prestressed concrete bearing the main tensile stress. The slope of the reinforcement curve gradually increases, exceeding the yield strain of about 360 kN. However, the curve does not increase sharply and keeps in the similar growth trends between load *P* and *Q*. This is because the steel strand has not reached the yield strain and can continue to bear the tensile stress, resulting in the stable growth of strain and deflection of non-prestressed reinforcement. It can be indicated that when the non-prestressed reinforcement yields, the beam does not fail and can still bear the normal load. At this time, the test beam is in the post-elastic stage. Afterload *Q*, the strain of steel increases sharply different from the growth trend between load *P* and *Q*, which manifests that the test beam enters the failure stage. Therefore, the strain curves of concrete and steel also have the mutation characteristics around characteristic loads, and the curve growth trends in the whole loading process have obvious characteristics according to each structural stressing the stage.

### 4.3. Reflection of Stress State Characteristics of Test Beam in Deflection

**Figure 5:**
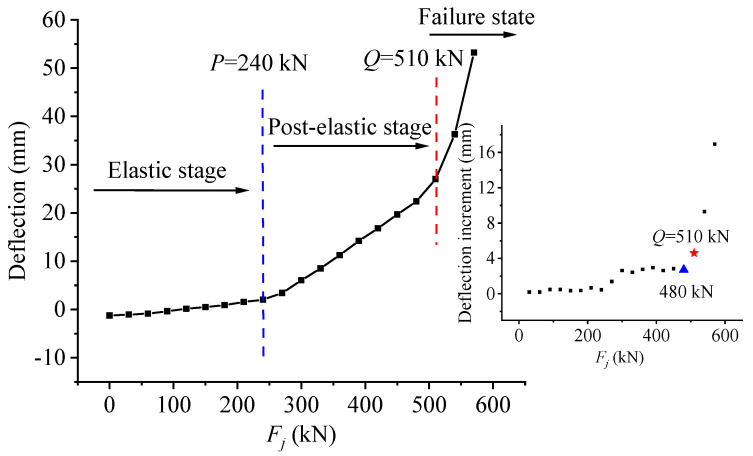
The load-deflection diagram of unbonded No. 4 beam (UP4).

As shown in Figure 5, the deflection curve of the test beam UP4 in the midspan section changes with the load. In order to reflect the deflection changes before and after all levels of loads, draw the deflection increment curve, and mark the characteristic points *P* and *Q* with blue and red dotted lines in the load-deflection diagram. It can be seen from Figure 5 that the two dashed lines divide the curve into three parts, which represent the three states of the test beam in the loading process, which is the same as the divided stage above. Because the partially prestressed concrete test beam has a certain camber value under the action of dead load and effective preload, the initial deflection of the test beam is negative before the external load is applied. With the increase of the applied load, the deflection under the beam produced by the external load is equal to the camber value produced by the preload, and they just offset each other. The deflection of the beam is zero, but the concrete stress at the edge of the tensile zone is not zero. Compared with the load-deflection curve, the deflection increment mutation characteristics can be observed more clearly, which can also verify the effectiveness of characteristic loads.

## 5. Analysis of Stress State of Test Beams with Different Design Parameters

### 5.1. Strain Energy Analysis of Test Beam Section under Different Working Conditions

To get the development trend of the test beam and the relationship among the characteristic loads, the design parameters of the test beams, and others, the test data of 12 test beams are compared and analyzed. As shown in Table 5, the respective characteristic loads *P* and *Q* values of the 12 test beams are summarized, and the *E_j_*-*F_j_* curves of 12 test beams are respectively drawn in two figures according to their bonded form of prestress (namely bonded or unbonded partially prestress).

**Figure 6:**
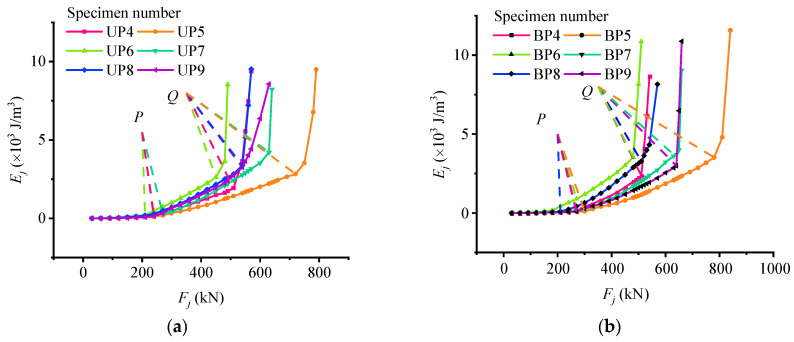
Strain energy density load curve: (**a**) strain energy load curve of unbonded partially prestress (UP) beam; (**b**) strain energy load curve of bonded partially prestress (BP) beam.

As shown in Figure 6, the variation characteristics of the 12 test beams are similar, that is, their respective characteristic loads can divide their respective *E_j_*-*F_j_* curve into three structural stressing state stages by the M-K standard. Combined with the differences in the design of the test beams in Table 1, it can be seen that the addition of non-prestressed or prestressed reinforcement to the unbonded test beams can improve their bearing capability and increase characteristic load values. Compared with beams 4 and 6 (or beams 5 and 7), it can also be found that the influence of prestressed reinforcement on bearing capacity is more obvious than that of non-prestressed reinforcement. In addition, the influence of prestressed steel strand reinforcement ratio on the deformation of the test beam is mainly reflected in the cracking of the test beam, that is, the higher the reinforcement ratio, the greater the flexural stiffness and deformation resistance of the test beam. After the non-prestressed steel bars yield, the prestressed steel strand can better control the weakening of bending stiffness and make the deformation recovery ability of the test beam better. However, the effect of the increase of concrete strength on the flexural stiffness of partially prestressed concrete beams is not significant.

### 5.2. Analysis of Deflection Influencing Factors

**Figure 7:**
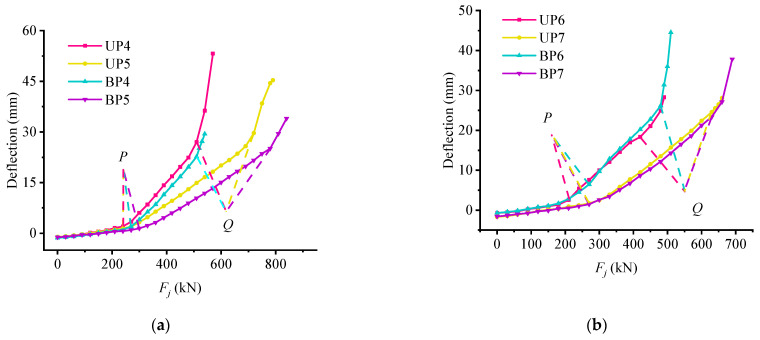
The influence of steel strand reinforcement ratio on mid-span deflection: (**a**) the influence of non-prestressed reinforcement ratio on deflection; (**b**) the influence of prestressed steel strand reinforcement ratio on the deflection.

**Figure 8:**
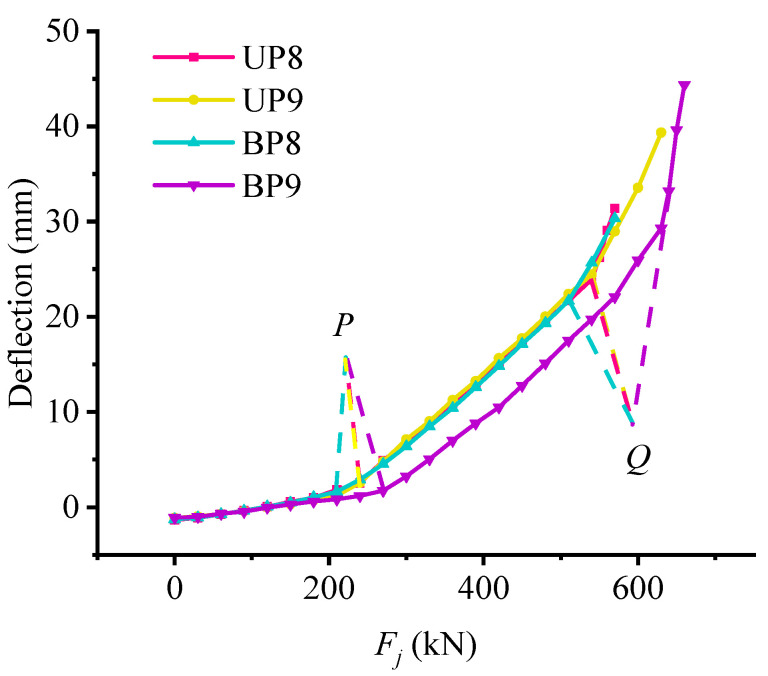
The influence of concrete strength on mid-span deflection (UP indicates unbonded partially prestress beam; BP indicates bonded partially prestress beam).

In order to study the influence of various design parameters on the mid-span deflection of the beam, Figure 7 and Figure 8 are drawn. As for beams BP4, BP5, UP4, and UP5, it can be found from Figure 7a that the mid-span deflections of both bonded and unbonded partially prestressed concrete beams are coincident in the uncracked stage of the test beam, namely, before their load *P*. After that, the curves separate, and the curve slope of the No. 5 beam with a larger non-prestressed reinforcement ratio is smaller than that of the No. 4 beam. Then, the second turning point of the No. 4 beam is first generated, and the deformation of the No. 4 beam is less than that of the No. 5 beam until destruction. This is because increasing the non-prestressed reinforcement ratio can improve the flexural stiffness of partially prestressed concrete beams after cracking and can effectively control the weakening of flexural stiffness. As shown in Figure 7b, the influence of the reinforcement ratio of prestressed steel strand on the mid-span deflection is shown. Before load *P*, the deflection curve of the test beam is relatively close, which indicates that the reinforcement ratio of the steel strand does not affect the initial bending stiffness. However, it can be found that the initial deflection of the UP7 and BP7 beams with higher prestressed steel strand reinforcement ratio is larger than that of UP6 and BP6 beams, and the different values and their increments begin to increase afterload *P*. Therefore, it can be considered that the influence of the reinforcement ratio of prestressed steel strand on the deformation of the partially prestressed concrete beam is mainly reflected in the cracking of the test beam, that is, the higher the reinforcement ratio, the greater the flexural stiffness, and the stronger the deformation resistance of the test beam. The weakening of bending stiffness can be better controlled, and the deflection growth is more stable. As for the influence of concrete strength on mid-span deflection shown in Figure 8, it can be found that the deflection curves of the four beams almost overlap each other before load *P*, but then the curve of the BP9 begins to increase by oneself. Therefore, increasing the concrete strength has no significant effect on the flexural stiffness of partially prestressed concrete beams, but it increases the deflection value at failure.

Combined with Figure 7 and Figure 8, comparing the deflection curves of bonded test beam and unbonded test beam, it can be found that the deflection curve of the test beam almost overlaps before load *P*, namely, in the uncracked stage of the test beam, and the bonding form of prestressed steel strand has no significant effect on the trend of deflection curve, but under the same load, the deflection of unbonded test beam is slightly larger.

## 6. Strain State Analysis Based on Strain Interpolation

### 6.1. Strain Field Interpolation Method Based on NSF Method

Generally, the experimental data of strain, stress, and displacement can characterize the mechanical properties of the structure, but due to various inevitable factors (limited measuring points, environmental factors and improper experimental operation, etc.), the experimental data are insufficient and cannot fully express the mechanical properties of the whole structure. In order to solve this problem, a numerical shape function method based on thin-plate spline interpolation is proposed [32,33]. This method can use the limited node data to simulate the data of the entire section.

**Figure 9:**
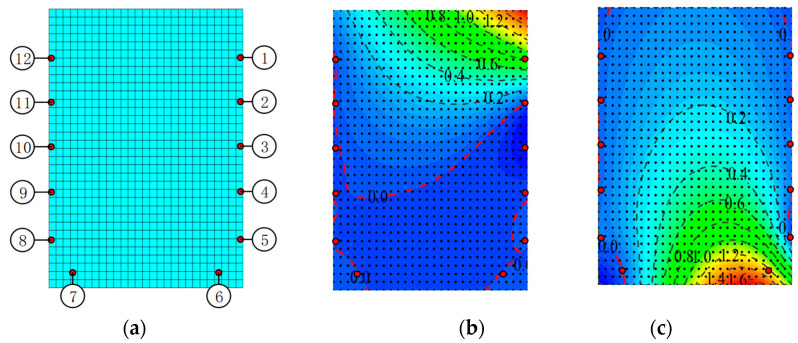
The finite element model of cross-section and profile of numerical shape function: (**a**) finite element model; (**b**) discrete weighting function N_1_; (**c**) discrete weighting function N_6_.

The NSF method is a new interpolation method combining traditional shape function and finite element simulation. In this paper, the mid-span section test is taken as an example to verify the effectiveness of the NSF method. As shown in Figure 9a below, the section of the model is modeled by ANSYS finite element software, and the section is reasonably divided into elements. The element type is shell 181 elements. Taking the 12 known measuring points on the rectangular section are numerical shape function points, then in the regularized coordinates (*x*, *y*), the coordinates (*x_j_,y_j_*) correspond to the coordinates of the *j*-th node. Points 1 to 12 represent the corresponding known strain *ε*_1_~*ε*_12._ Based on reasonable section element division, combined with the construction conditions of the numerical shape function, the numerical shape functions N_1_~N_12_ can be obtained according to the static simulation analysis. Taking N_1_ as an example, after static calculation, we extract function values at nodes from the FE model to obtain N_1_, as shown in Figure 9b. Similarly, the numerical shape function N_6_ obtained is also shown in Figure 9c. Therefore, without considering large deformation or elastic plasticity, the corresponding interpolation strain field is constructed by Castiliano’s law, and the simulation results are linearly superimposed to obtain the strain field D of the whole interface, that is

(9) $$D=\sum_{i=1}^{m}\varepsilon_{i}N_{i},N_{i}=\left[N_{i}\left(x_{1},y_{1}\right),N_{i}\left(x_{2},y_{2}\right),\ldots N_{i}\left(x_{j},y_{j}\right),\ldots N_{i}\left(x_{n},y_{n}\right)\right],$$

where N*_i_* represents the discrete numerical shape function at the *i*-th control point, N*_i_* (*x_j_,y_j_*) represents the function value at the node (*x_j_,y_j_*) in the coordinate system (*x*, *y*), and n is the total number of nodes. *ε_i_* represents the strain value of a known measuring point, and D is the strain field of the entire section, which can be regarded as the sum of the strains of all nodes on the entire section. 

It can be seen from the above that, combined with traditional shape function and finite element simulation, the NSF method can relatively accurately estimate the experimental data of the ensemble’s position, especially the area that is difficult to measure. For example, as shown in the figure above, the experimental data is strain. These limited data on the cross-section can be expanded by the NSF method to obtain the strain value on each element node. Then, according to the constitutive relation of the material, the corresponding stress data can be obtained. The stress–strain curve of uniaxial tension in the constitutive relation of concrete used in this test is shown in the following:

(10) $$\sigma =(1-d_{t})E_{c}\varepsilon ,$$

(11) $$d_{t}=\{\begin{array}{cc} 1-\rho_{t}[1.2-0.2x^{5}]\;, & x\leq 1\\ 1-\frac{\rho_{t}}{\alpha_{t}{\left(x-1\right)}^{1.7}+x}, & x>1 \end{array},$$

where *x* = *ε*/*ε_t,r_*. *ρ_t_* = *f_t,r_*/*E_c_ε_t,r_*. *f_t,r_* represents the representative value of uniaxial tensile strength of concrete (MPa); *α_t_* represents the parameter of the descending section of the stress-strain curve of concrete under uniaxial tension; *ε_t,r_* represents the peak tensile strain of concrete corresponding to the representative value of uniaxial tensile strength; and *d_t_* represents the damage evolution parameter of concrete under uniaxial tension. The stress-strain curve of concrete under uniaxial compression is shown in the following:

(12) $$\sigma =(1-d_{c})E_{c}\varepsilon ,$$

(13) $$d_{c}=\{\begin{array}{cc} 1-\frac{\rho_{c}n}{n-1+x^{n}}, & x\leq 1\\ 1-\frac{\rho_{c}}{\alpha_{c}{\left(x-1\right)}^{2}+x}\;, & x>1 \end{array},$$

where *x* = *ε*/*ε_t,r_*. *ρ_t_* = *f_t,r_*/*E_c_ε_t,r_*. *n* = *E_c_ε_t,r_/*(*E_c_ε_t,r_* − *f_t,r_*); *f_c,r_* represents the representative value of uniaxial compressive strength of concrete (MPa); *α_c_* represents the parameter of the descending section of the stress–strain curve of concrete under uniaxial compression; *ε_c,r_* represents the peak tensile strain of concrete corresponding to the representative value of uniaxial compressive strength; and *d_c_* represents the damage evolution parameter of concrete under uniaxial compression. Then, the sectional internal forces can also be obtained by integration, and as for the test beams, the in-plane bending moments are their main internal forces, which can be calculated by the following:

(14) $$M_{j}=\int_{A}\sigma ydA=\sum_{A}\sigma_{ij}y_{i}A_{i},$$

### 6.2. Error Analysis of the NSF Method

In order to verify the fitting degree between the test data and the data obtained by NSF method, the data of No. 2 and No. 5 measurement points of test beam UP4 are selected for error analysis. By comparing these values with the actual measured values, the error of the *i*-th point under the *j*-th load step is calculated by the following formula.

(15) $$\delta_{ij}=\left|\frac{\varepsilon_{ij}^{t}-\varepsilon_{ij}^{e}}{\varepsilon_{ij}^{e}}\times 100\%\right|,$$

where $\delta_{ij}$ is the error of *i*-th point under the *j*-th load steps; $\varepsilon_{ij}^{t}$ represents the interpolating strain of *i*-th point under the *j*-th load steps; and $\varepsilon_{ij}^{e}$ represents the experimental strain at the *i*-th point under the *j*-th load steps.

**Figure 10:**
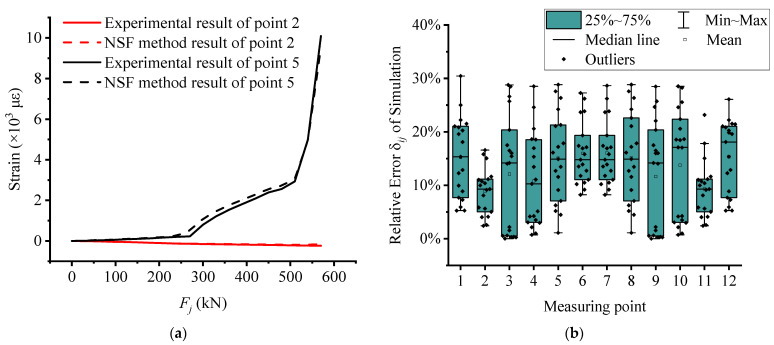
The error analysis of the NSF method: (**a**) comparison between experimental strain and interpolated strain of unbonded No. 4 beam; (**b**) box plot of simulation relative error of unbonded No. 4 beam.

As shown in Figure 10a, the strain curves of measuring points 2 and 5 are obtained by the NSF method. The results show that the curves at the same point coincide, and the fitting degree is good. In order to more clearly reflect the errors of all measurement points, the simulated relative error block diagram shown in Figure 10b is drawn. Since the measuring points on the cross-section are symmetrically arranged, only the percentage of strain error of measuring points 1–6 can be analyzed. It can be found that the boxes of measurement points 3 and 4 are relatively long, which indicates that the fluctuation of interpolation data of these two measurement points is greater than that of other measurement points. The boxes of measuring points 2 and 6 are small, and the finish line is short, which reflects the relative concentration of data at these two measuring points. In short, the NSF method with sufficient accuracy can expand the test data and further study the structural performance of the test beam.

### 6.3. Analysis of Strain Field/Stress Field

**Figure 11:**
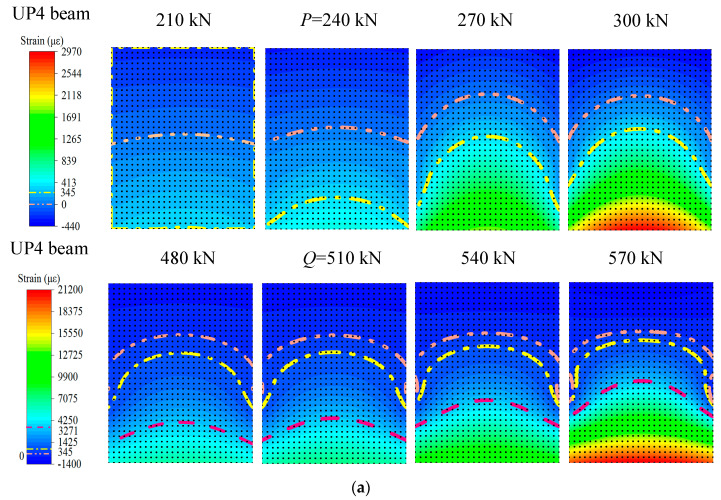

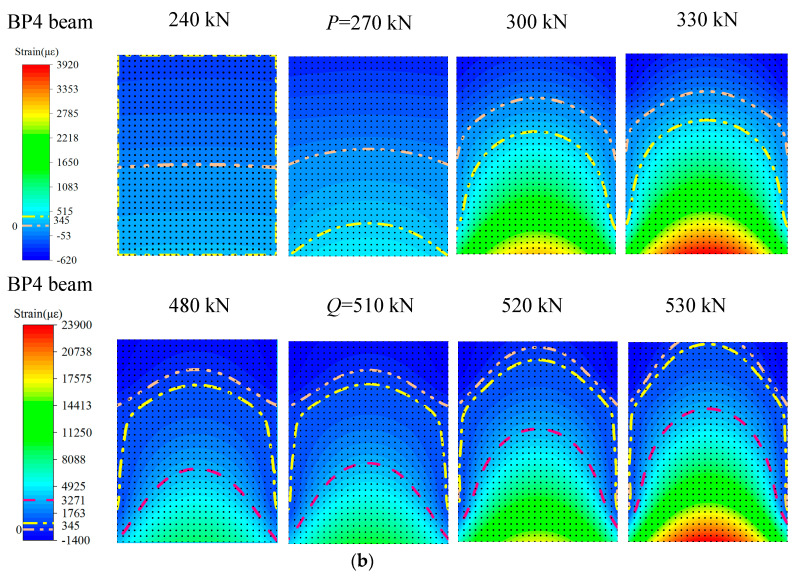
The strain fields diagrams of unbonded No. 4 beam (UP4): (**a**) the strain fields around characteristic point *P*; (**b**) the strain fields around characteristic point *Q*.

The limited experimental strain data can only reflect the strain distribution and development trend of each measuring point. Therefore, the NSF method is used to obtain the section strain/stress data, that is, the section strain/stress field. The strain fields of beams UP4 and BP4 are drawn in Figure 11, and the strain of 0 με, the ultimate tensile strain of concrete (345 με) and the yield strain of the non-prestressed steel bar (3271 με) are respectively marked by different dotted lines of different colors.

As can be seen from Figure 11a, when it does not reach load *P*, the neutral axis (namely, the axis of 0 με) is located on the centroid axis of the whole structure. When the applied load reaches the characteristic load *P*, the neutral axis of the structure moves up. This is because the upper part of the beam is compressed, and the lower part is tensioned under the effective pretension stress of the prestressed steel strand. The bottom edge of the test beam reaches the peak tensile strain and cracks gradually appear. As the external load continues to increase, the concrete cracks expand upward, and the lower edge concrete gradually withdraws from the work. On the contrary, prestressed reinforcement and tensile reinforcement bear a large amount of load. By comparing the strain field changes of UP4 and BP4 beams before and after the characteristic load *P*, it is found that the neutral axis and concrete ultimate tensile strain positions of both UP4 and BP4 beams are the same before the characteristic load *P*, but after the characteristic load *P*, the neutral axis and concrete ultimate tensile strain positions of BP4 beam are higher. The reason is that the UP4 beam is a partially prestressed concrete beam with bonding. There is the adhesive force between prestressed reinforcement and concrete in the beam, and the strain of prestressed reinforcement changes synchronously with that of concrete.

As can be seen from Figure 11b, before and after the characteristic load *Q*, the ultimate tensile strain line and neutral axis of concrete do not change significantly, while the yield strain line of reinforcement changes significantly. As the prestressed beam gradually loses the tensile effect of the test, most of the non-prestressed beam will bear the tensile load. With the continuous increase of load, the non-prestressed reinforcement reaches the yield strain, and then its strain increases sharply. Compared with the UP4 beam, the ultimate tensile strain line and neutral axis of the BP4 beam change significantly after characteristic load *Q*. This is because after the structural failure of the BP4 beam, the bond between prestressed reinforcement and concrete fails, and the bearing capacity of non-prestressed tensile reinforcement is enhanced.

**Figure 12:**
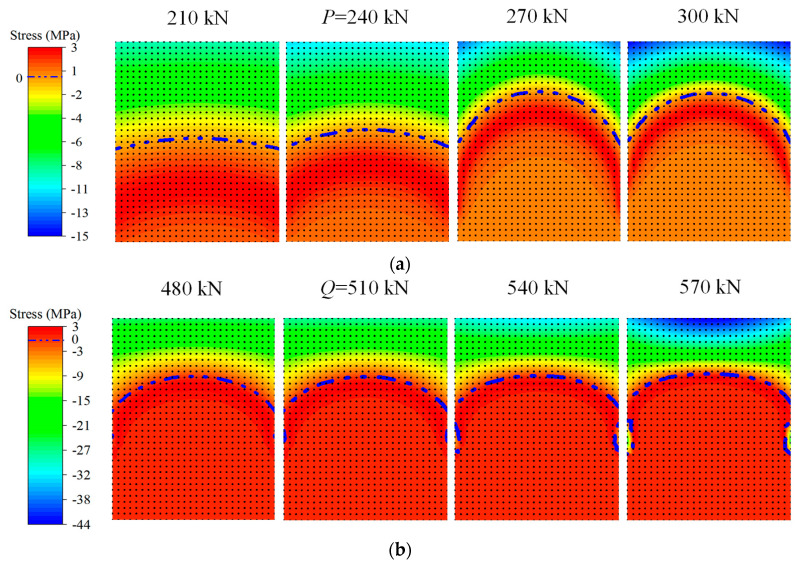
The stress fields of unbonded No. 4 beam (UP4): (**a**) the stress fields around characteristic point *P*; (**b**) the stress fields around characteristic point *Q*.

The stress fields around characteristic loads *P* and *Q* are drawn in Figure 12. It can be seen that due to the development of elastic deformation after the concrete in the tensile area, the tensile stress of the concrete at the tensile edge reaches the tensile strength of the concrete, and cracks are about to appear. When the load reaches a certain strength, the first crack appears in the weakest section of concrete tensile strength. As the load continues to increase, the number of cracks in the section increases, and the stress on the section redistributes. When the concrete in the tension area fails, its original tension is transferred to the non-prestressed reinforcement. Due to the cracking of concrete in the tensile area, almost all the tensile force is borne by non-prestressed reinforcement. Only part of the concrete near the neutral axis is not cracked, and the tensile force is very small. When the non-prestressed steel bar yields, the tensile strain of the steel bar increases rapidly, but the stress of the steel bar remains unchanged at the yield strength. The compressive axial stress of concrete continues to increase rapidly, and the compressive stress of concrete continues to increase.

### 6.4. Analysis of Internal Forces Calculated by the NSF Method

**Figure 13:**
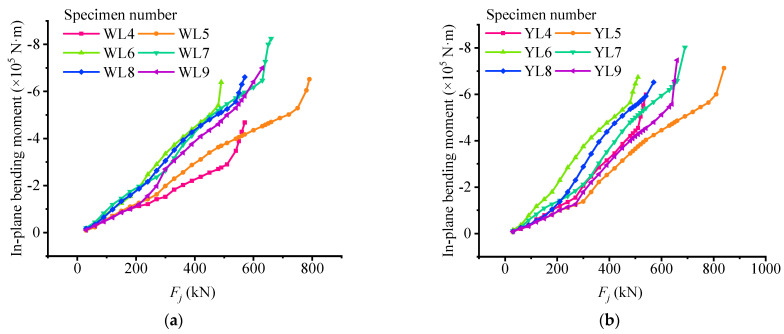
In-plane bending moment diagram: (**a**) in-plane bending moment of unbonded partially prestress beam; (**b**) in-plane bending moment of bonded partially prestress beam.

Under the load, because the out-of-plane deformation of the test beam is restrained, it mainly bears the in-plane bending moment. Figure 13 is the in-plane bending moment diagram of the mid-span section of 12 test beams. It can be seen that all beams have similar change trends around their respective characteristic loads, and the curves have obvious mutation characteristics. Under the failure load, the in-plane bending moment of the bonded partially prestressed concrete beam is usually greater than that of an unbonded partially prestressed concrete beam. Under the same load, the bending moment of the C40 concrete beam is greater than that of the C60 concrete beam. In addition, compared with the beams 5 and 7 (or beams 4 and 6), it can be found that compared with reinforcement, prestressed reinforcement has a greater impact than non-prestressed one on the in-plane bending moment. The additional bending moment is provided by the preloading stress, which applies the pre-compression stress of the concrete at the lower edge of the beam in the initial state. When the ratio of prestressed reinforcement is higher, the value of the cracking moment caused will be larger, and the cracking moment of the concrete beam will be larger.

## 7. Conclusions

In this paper, through the structural stressing state theory and numerical shape function method, the mechanical characteristics of partially prestressed concrete beams with 600 MPa non-prestressed reinforcement under static load are revealed. The conclusions are:

1. Generalized strain energy density is introduced to present the structural stressing state of the whole test beams and each beam’s two characteristic loads, namely, post-elastic bound load *P* and failure load *Q*, can be judged by the Mann–Kendall criterion, which can also divide their structural stressing state into three stages, namely, linear elastic, post-elastic, and failure stage. 

2. The numerical shape function method is proposed to interpolate the sectional strain, and the strain and stress fields and internal forces can be obtained to analyze the changing characteristics of the test beams’ structural stressing state. The mutation characteristics of strain, deflection, strain/ stress fields, and the in-plane bending moment around the loads *P* and *Q* can not only also verify the correctness and efficiency of the Mann-Kendall criterion.

3. Compared with the ordinary concrete beams, partially prestressed concrete beams with 600 MPa non-prestressed reinforcement can well control the crack propagation and deflection development of the test beam after yield. The non-prestressed and prestressed reinforcements work together to give full play to the strength of the reinforcement and can effectively improve the bending capacity and deformation capacity.

4. By configuring the partially prestressed concrete beams with 600 MPa non-prestressed reinforcement, the weakening process of the bending stiffness of the test beam can be effectively controlled, and characteristic loads can be improved by increasing the reinforcement ratio of prestressed reinforcement or the strength of concrete. In addition, the bending stiffness of the test beam after cracking can be effectively improved by increasing the reinforcement ratio.

In this paper, based on the test, the mechanical characteristics of partially prestressed concrete beams with 600 MPa non-prestressed reinforcement are explored deeply, and the characteristic loads of the test beam are accurately found. We hope that these methods and results can promote the development of structural design and practice of high-strength reinforced concrete beams.

## Figures and Tables

**Table 1:** Design parameters of each test beam.

Member Number	Concrete Strength Grade	Quantity of Prestressed Reinforcement	Non-Prestressed Tensile Reinforcement	
			Number	Steel Strength (MPa)
BP1/UP1	C40	3	3	400
BP2/UP2	C50	3	3	500
BP3/UP3	C50	3	3	600
BP4/UP4	C50	3	2	600
BP5/UP5	C50	3	4	600
BP6/UP6	C50	2	3	600
BP7/UP7	C50	4	3	600
BP8/UP8	C40	3	3	600
BP9/UP9	C60	3	3	600

Note: (1) The number of strand wires in each channel of the prestressed reinforcement in the test beam is 1. (2) The distance of the tensile reinforcement from the bottom edge of the test beam is 49 mm; the distance of the prestressed reinforcement from the bottom edge of the test beam is 98 mm, and the rest of the longitudinal reinforcement is the erection bar. (3) The bonded beam is named the BP beam and the unbonded beam is named the UP beam.

**Table 2:** Measured mechanical properties of non-prestressed tensile reinforcement.

Steel Type	*D_f_* (mm)	*f_y_* (MPa)	*f_st_* (MPa)	*E_s_* (GPa)
400 MPa	18	439	613	200
500 MPa	18	552	711	200
600 MPa	18	654	824	200

Note: *D_f_*—Diameter of non-prestressed reinforcement; *f_y_*—The measured yield strength of non-prestressed reinforcement; *f_st_*—The measured value of ultimate strength of non-prestressed reinforcement; *E_s_*—The elastic modulus of non-prestressed reinforcement.

**Table 3:** Mechanical properties of prestressed strand.

Batch	*D_n_* (mm)	*f_py_* (MPa)	*E_p_* (GPa)
1	15	1885	190
2	15	1885	195
3	15	1864	197
Specification		≥1860	195

Note: *D_n_*—Diameter of prestressed steel bar; *f_py_*—Tensile strength of prestressed steel bars; *E_p_*—Modulus of elasticity of prestressed steel bars.

**Table 4:** Measured mechanical properties of concrete.

Concrete Strength Grade	*f_cu_* (MPa)	*f_t_* (MPa)	*E_c_* (GPa)
C40	47.3	2.9	34
C50	54.2	3.0	35
C60	67.4	3.3	36

Note: *f_cu_*—Compressive strength of concrete cube test block; *f_t_*—Axial tensile strength of concrete; *E_c_*—Elastic modulus of concrete.

**Table 5:** Characteristic loads of test beams.

Member Number	Characteristic Load *P* (kN)	Characteristic Load *Q* (kN)	Member Number	Characteristic Load *P* (kN)	Characteristic Load *Q* (kN)
UP4	240	510	BP4	270	510
UP5	240	720	BP5	300	780
UP6	210	480	BP6	270	480
UP7	270	630	BP7	270	630
UP8	240	540	BP8	210	510
UP9	240	540	BP9	270	640

## Data Availability

Data are available on request to the authors.

## Nomenclature

*E_ij_*	the GSED value of the i-th point under the j-th load
*ε_ij_*	the strain value of this point
*E_j_*	the GSED and value of each point under the action of the j-th load
*E*_max_	the maximum GSED and value in the whole process
*E_j,_* _norm_	all measuring points reflect the stress state of the structure under each load step F_j_
*φ* ^s^	stands for steel strand
*D_f_*	Diameter of non-prestressed reinforcement
*f_y_*	The measured yield strength of non-prestressed reinforcement
*f_st_*	The measured value of ultimate strength of non-prestressed reinforcement
*E_s_*	The elastic modulus of non-prestressed reinforcement
*D_n_*	Diameter of prestressed steel bar
*f_py_*	Tensile strength of prestressed steel bars
*E_p_*	Modulus of elasticity of prestressed steel bars
*f_cu_*	Compressive strength of concrete cube test block
*f_t_*	Axial tensile strength of concrete
*E_c_*	Elastic modulus of concrete
*f_t,r_*	the representative value of uniaxial tensile strength of concrete
*α_t_*	the parameter of the descending section of the stress-strain curve of concrete under uniaxial tension
*ε_t,r_*	the peak tensile strain of concrete corresponding to the representative value of uniaxial tensile strength
*d_t_*	represents the damage evolution parameter of concrete under uniaxial tension
*f_c,r_*	the representative value of uniaxial compressive strength of concrete
*α_c_*	the parameter of the descending section of the stress-strain curve of concrete under uniaxial compression
*ε_c,r_*	the peak tensile strain of concrete corresponding to the representative value of uniaxial compressive strength
*d_c_*	represents the damage evolution parameter of concrete under uniaxial compression
